# Uncovering structural variants in Creole cattle from Guadeloupe and their impact on environmental adaptation through whole genome sequencing

**DOI:** 10.1371/journal.pone.0309411

**Published:** 2024-08-26

**Authors:** Slim Ben-Jemaa, Mekki Boussaha, Nathalie Mandonnet, Philippe Bardou, Michel Naves

**Affiliations:** 1 INRAE, ASSET, 97170, Petit-Bourg, France; 2 Institut National de la Recherche Agronomique de Tunisie, Laboratoire des Productions Animales et Fourragères, Université de Carthage, 2049, Ariana, Tunisia; 3 Université Paris-Saclay, INRAE, AgroParisTech, GABI, 78350, Jouy-en-Josas, France; 4 GenPhySE, Université de Toulouse, INRA, Ecole Nationale Vétérinaire de Toulouse (ENVT), 31320, Castanet-Tolosan, France; 5 Sigenae, INRAE, 31320, Castanet-Tolosan, France; National Bureau of Animal Genetic Resources, INDIA

## Abstract

Structural variants play an important role in evolutionary processes. Besides, they constitute a large source of inter individual genetic variation that might represent a major factor in the aetiology of complex, multifactorial traits. Their importance in adaptation is becoming increasingly evident in literature. Yet, the characterization of the genomic landscape of structural variants in local breeds remains scarce to date. Herein, we investigate patterns and gene annotation of structural variants in the Creole cattle from Guadeloupe breed using whole genome sequences from 23 bulls representative of the population. In total, we detected 32821 ascertained SV defining 15258 regions, representing ~ 17% of the Creole cattle genome. Among these, 6639 regions have not been previously reported in the Database of Genomic Variants archive. Average number of structural variants detected per individual in the studied population is in the same order of magnitude of that observed in indicine populations and higher than that reported in taurine breeds. We observe an important within-individual variability where approximately half of the detected structural variants have low frequency (MAF < 0.25). Most of the detected structural variants (55%) occurred in intergenic regions. Genic structural variants overlapped with 7793 genes and the predicted effect of most of them is ranked as “modifier”. Among the structural variants that were predicted to have a high functional impact on the protein, a 5.5 Kb in length, highly frequent deletion on chromosome 2, affects *ALPI*, a gene associated with the interaction between gut microbiota and host immune system. The 6639 newly identified structural variants regions include three deletions and three duplications shared by more than 80% of individuals that are significantly enriched for genes related to tRNA threonylcarbamoyladenosine metabolic process, important for temperature adaptation in thermophilic organisms, therefore suggesting a potential role in the thermotolerance of Creole cattle from Guadeloupe cattle to tropical climate. Overall, highly frequent structural variants that are specific to the Creole cattle population encompass olfactory receptor and immunity genes as well as genes involved in muscle tone, muscle development and contraction. Beyond mapping and characterizing structural variants in the Creole cattle from Guadeloupe breed, this study provides valuable information for a better understanding of the potential role of chromosomal rearrangements in adaptive traits in cattle.

## Introduction

Structural variants (SV) are large DNA rearrangements (> 50 bp in length) affecting an individual’s genome. They can be balanced and show no specific loss or gain of genetic material, such as inversions of a genetic fragment or translocations of a stretch of DNA within or between chromosomes, or they can be unbalanced, where a part of the genome is lost (insertions/deletions) or duplicated (duplications). In the latter case, structural variants are also termed copy number variation (CNV) [1]. Structural variants are ubiquitous and affect a greater fraction of the genome than single nucleotide polymorphisms (SNPs) [2]. They have been extensively studied in humans where they have been shown to constitute potent phenotypic modifiers that act through multiple mechanisms, such as altering gene dosage, disrupting regulatory elements, generating fusion proteins or unmasking of recessive alleles, thus causing several human disorders [3]. Structural variants are also considered as an important driver of evolution that may enable rapid adaptation to environmental stressors in animals and plants [4–6]. SV are poorly characterized in livestock [7]. Yet, several studies highlighted their influence on several phenotypic traits [8–10] in several domestic animals. In cattle, SV are responsible for variation in coat colour [11], and several complex traits, including milk production, fertility, and other traits [8,12–14]. The majority of SV studies carried out in cattle were SNP-array based [15]. It has only been recently that studies based on second- and third-generation sequencing technologies begin to emerge for local breeds [16–18]. Regardless of the sequencing technology used, a significant proportion of the identified SV in cattle was shown to be breed-specific, suggesting a potential association with differences in adaptation, health, and production traits [19–21]. Breed-specific SV can potentially store important information on the genomic architecture of adaptive traits. This is particularly true for small local breeds that have been exposed to various selective pressures in a given environment.

Identifying SV associated with environmental adaptation would be of utmost importance in the climate change context, particularly for cattle where high-producing breeds, more sensitive to heat stress and less adapted to emerging pathogens are replacing locally adapted populations.

Livestock species have been introduced in Latin America and the Caribbean after the discovery of the new world by Colombus, in the 15th century. The first specimen introduced came from the Iberian Peninsula, but afterwards, between the 16th and 18th century, animals from other origins were brought, following the complex history of colonization and human settlement in the region [22]. These complex migratory and admixture events, combined with natural selection and traditional usage, led to the constitution of the different Creole breeds, presenting specific features according to their location. In the Caribbean, under the French influence, important migrations followed the slave-trade route from Western Africa, with probably both small shorthorn taurine cattle and indicine admixed populations, as attested by historical data [23].

**Fig 1:**
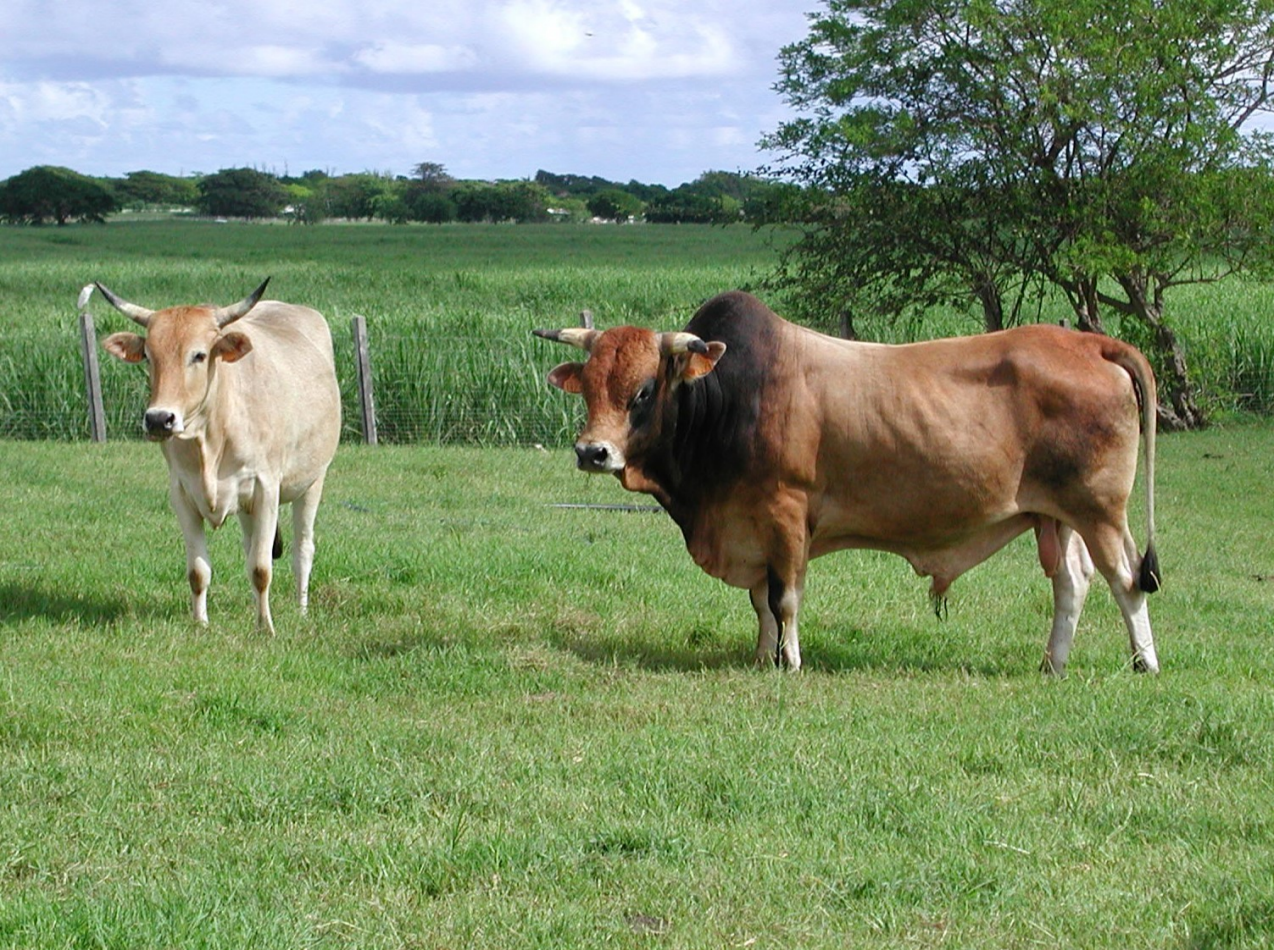
Cow and sire representative of the Creole cattle from Guadeloupe. The hump and the dewlap in the males are well-developed.

Creole cattle in the Guadeloupe island (GUA) is an admixed breed resulting from a three-way admixture between African taurine, European taurine and zebu (Fig 1). GUA individuals show good production and reproduction abilities under warm climate and harsh conditions and are known to be resistant to several endemic tick-borne diseases in the island [24]. Recently, through whole-genome sequence analysis of 23 GUA individuals, we provided a detailed examination of genetic variation and we identified several candidate regions potentially associated with specific adaptive features in the GUA genome [25]. In order to capture the different axes of diversity in GUA population, we herein report for the first time, a genome-wide characterization of structural variants derived from whole-genome resequencing data in these 23 GUA genomes. We also highlighted the potential role of the identified chromosomal structural variation in local adaptation of GUA population.

## Materials and methods

### Animal ethics statement

Blood collection was done according to good practices recommended for identification of sires for paternity checking in France. This study was approved by the scientific committee of the Metaprogramme SELGEN of INRAE.

### Sample information and genome sequencing

Twenty three Creole bulls representative of the INRA nucleus herd in Guadeloupe were selected for the purpose of this study. Ten out of them are sons of founder sires of the experimental flock while the remaining thirteen animals are unrelated sires (based on genealogical records and sampling sites) chosen from the local stock of Guadeloupe. Genomic DNA was extracted from whole-blood and semen samples collected between 1995 and 2015. Paired-end libraries with insert size of 500 bp were constructed for each individual and sequenced using the HiSeq 3000 platform (Illumina) in the Genome et Transcriptome (GeT) GénoToul platform (Toulouse, France), following the manufacturer’s protocol.

### Sequence alignment and SV discovery

Quality control of raw sequence reads was performed using the fastQC software v.0.11.7 [26]. Trimmomatic-0.36 [27] was used to remove Illumina adapter sequences, low-quality bases and artefact sequences. Filtered sequences were then mapped against the bovine reference genome (ARS-UCD1.2) using the Burrows-Wheeler Alignment tool (bwa mem v.0.7.17) [28] with default parameters. The resulting SAM files were then converted to BAM format, sorted, and indexed using SAMtools [29]. PCR duplicates were removed using the MarkDuplicates tool from Picard version 1.88 (http://broadinstitute.github.io/picard). Only properly paired reads with a mapping quality of at least 30 were kept. Local realignment was performed using two GATK (Genome Analysis Toolkit) version -3.8-1-0-gf15c1c3ef modules, RealignerTargetCreator and IndelRealigner. We used three SV callers, Pindel (v.0.2.5), LUMPY (v.0.2.13) and DELLY (v.0.7.8). We developed a custom pipeline combining the detection results of the three aforementioned SV-finding algorithms. We retained only SV identified by at least two callers and having a length between 50 bp and 5 Mb because structural variants identification tools relying solely on a single principle were shown to generate many false positive calls [30,31]. According to their frequency within the GUA sample, we have defined four SV categories: singletons (detected in one individual), low frequency (detected in less than 25% of individuals), common (detected in more than 25% and less than 80% of individuals) and highly frequent (detected in more than 80% of individuals). The R package karyoploteR [32] was used to display SV distribution across 30 chromosomes (29 autosomes and the X chromosome). To identify novel structural variations that had not been discovered so far, the genomic regions defining the SV detected in the present study were intersected with known structural variations reported in cattle from Database of Genomic Variants archive (DGVa) using the function ‘intersect’ from BEDtools [33]. For this purpose, we defined non-overlapping SV regions for the three types of SV and we compared these regions to the 9277 SV regions reported in the DGVa database.

### Functional impact of structural variants

Ensembl Variant Effect Predictor (http://www.ensembl.org/info/docs/tools/vep/index.html)(VEP) was used to provide a prediction for each transcript with which the common and highly frequent SV overlap (those whose MAF > 0.25). VEP was used to determine the location of the SV (e.g. intronic, intergenic, upstream of a transcript, in coding sequences, in regulatory regions) as well as the predicted effect of SV on the protein (e.g. stop lost, frameshift). VEP also provides an impact rating (high, moderate, low, and modifier) indicating the severity of the consequences.

Functional enrichment analysis was performed using the online tool DAVID version 6.8 (Database for Annotation, Visualization and Integrated Discovery, https://david.ncifcrf.gov/). DAVID uses thousands of annotation terms in several annotation categories, such as Gene Ontology (GO), Biological Process, GO Molecular Function and InterPro Domains. An adjusted Benjamini-corrected p-value of 0.05 was used as the criterion for statistical significance of over-enrichment of genes in one of the categories.

## Results and discussion

### Sequencing, SV discovery, and validation

A total of 7,543,644,154 clean reads were generated after sequencing the complete genome of the 23 samples, representing an average depth of ~16.35 fold (min depth = 9.3; max depth = 23.77). The average alignment rate against the *Bos Taurus* reference genome (ARS-UCD1.2), for the different individuals varied from 94.93% to 96.51% with an average of 95,7% (S1 Table). Three classes of SV: deletions (DEL), duplications (DUP) and inversions (INV) with a size ranging between 50 bp and 5 Mb were detected. Among the three callers, Pindel exhibited the highest detection sensitivity for deletions and duplications while DELLY identified much more inversions. LUMPY, for its part, called a lower number of SVs for all the three types of structural variants (Table 1). In total, 69% and 87% of the deletions (those at a minimum overlap identity of 90%) identified by Pindel and LUMPY, respectively were also detected by DELLY but this latter detected only 36% and 49% of the deletions, identified by Pindel and LUMPY, respectively. A high percentage, 94%, of the duplications identified by LUMPY were also identified by DELLY, but only 42% of those identified by DELLY were also identified by LUMPY. Also, we found that 59% of the duplications detected by DELLY were identified by Pindel and that 69% of those identified by Pindel were revealed by DELLY. In total, 92% of the inversions detected by Pindel were also identified by DELLY but only 53% of the inversions identified by DELLY were detected by Pindel.

**Table 1:** Performance of the three SV callers in detecting different SV types within the 23 Creole cattle individuals.

SV caller	SV type	Count
Pindel	DEL	1,089,575
DELLY	DEL	201,233
LUMPY	DEL	147,582
Pindel	DUP	69,763
DELLY	DUP	35,385
LUMPY	DUP	10,269
Pindel	INV	30,299
DELLY	INV	49,726
LUMPY	INV	1,342

In total, we identified 32,821 ascertained SV defining 15,258 regions with at least 2 software from the 23 GUA animals. Although combining multiple callers is recommended for a higher detection accuracy of structural variants [34], it should be outlined that SV detection in the present study is likely to be altered by several factors that are related to the sequencing technology used in the present study (short read sequencing) and to the threefold ancestry of the Creole cattle from Guadeloupe. For instance, insertions longer than short reads are expected to be easily missed because they cannot align correctly with the reference genome [35]. Likewise, the use of linear reference genomes causes reference allele bias that affects genetic variation detection tools [36]. Hence, we expect that the detection of SVs that are from African taurine and indicine origin in the genome of GUA, woud be affected when aligning the mapped reads to the Hereford reference genome assembly ARS-UCD1.2. Clearly, using a personalized reference genome of GUA should improve reliability of structural variation calls.

### SV distribution and statistics

**Fig 2:**
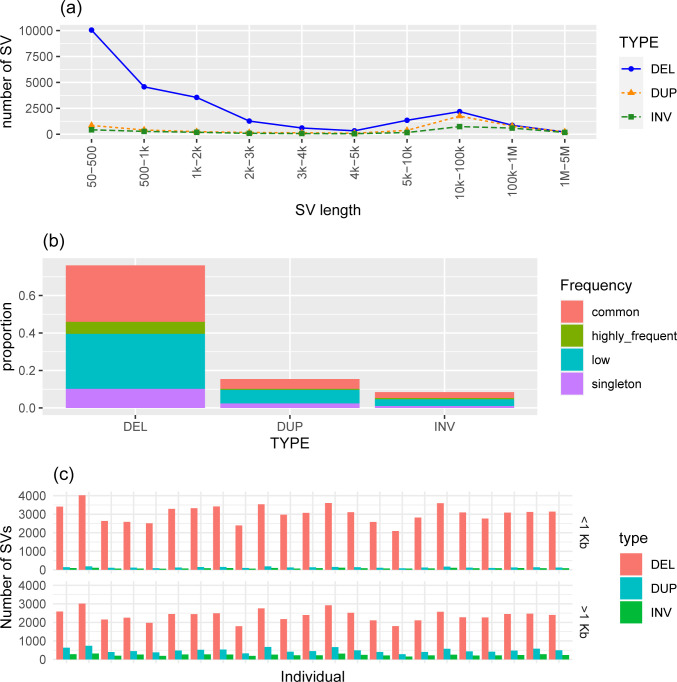
Statistics and individual distribution of structural variations (SVs) in the Creole cattle from Guadeloupe sample. (a) distribution of length for each SV type, (b) frequency of SVs by type. Four categories are defined: Singletons (detected in one individual), low frequency (detected in less than 25% of individuals), common (detected in more than 25% and less than 80% of individuals) and highly frequent (detected in more than 80% of individuals), (c) SV distribution among the 23 sequenced animals. The upper part shows SV < 1 Kb, while the bottom part shows SV lengths > 1 Kb.

In accordance with previous studies [37–39], we observe an abundance of deletions (76% of the total SV detected in the present study) compared to duplications and inversions. On the other hand, deletions have a similar range of total length to duplications (Table 2) which is due to the fact that most of the identified deletions (81.56%) were <5 kb in length, whereas more than half (~ 55%) of duplications and inversions are of large size (10 Kb <size<5 Mb) (Fig 2A). This agrees with previous findings reporting a possible correlation between SV type and size.

**Table 2:** Statistics of the SV number detected by at least two callers for the 23 GUA animals.

	Number of SV	Total length (Mb)
DEL	24981	792.46
DUP	5071	767.56
INV	2769	567.23
Total	32821	2127.25

Approximately half (~ 54%) of all detected SV have low frequency (detected in less than 6 individuals; Fig 2B) and covered 1.214 Gb of the chromosomes of the ARS-UCD1.2 assembly. About a quarter of these were detected in a single individual. This is consistent with previous studies indicating that high SV diversity exists among different cattle individuals [40,41]. Accordingly, we also observe an important within-individual variation of high-confidence SV. Their number ranged between 2092 and 4002 (average = 3051 ± 455) for deletions < 1 Kb, 1793 and 3009 (2362 ± 308) for deletions > 1 Kb, between 84 and 187 (average = 131 ± 28) for duplications < 1 Kb, 282 and 736 (average = 488 ± 114) for duplications > 1 Kb, and between 61 and 115 (average = 86 ± 16) for inversions < 1 Kb, 155 and 318 (average = 242 ± 40) for inversions > 1 Kb (Fig 2C). We observe, on average, 6360 SV per GUA individual which is in the same order of magnitude of African and Indian zebu but goes well beyond the values previously reported in European and African taurine breeds [38]. Part of the differences between GUA on one hand and taurine European and African populations on the other hand may be due to population structure. Indeed, the three-way admixture of the GUA genome [24] of which more than one third has an indicine origin is likely to be behind the high number of observed SV. Several studies have reported a higher SV in indicine than in taurine breeds which is consistent with the known breed divergence and history [42,43]. The origin of the indicine ancestry in GUA population appears controversial. Some historical evidences [23] relate introductions of West African cattle in Guadeloupe between the 16th and 18th century, and it is possible that African zebu as well as African taurine have been introduced. The alternate possibility of a recent introduction of Indian zebu in Guadeloupe at the end of the 19th century (as in the other regions of America) has not been documented [24]

A small portion (7.6%) of the detected SV is present in more than 80% of the GUA individuals (Fig 2B). Chromosomal distribution of common and highly frequent SV regions and having a size > 1 Kb was not uniform and varied according to the SV type. Chromosome 25 has the lowest number of deletions and inversions (47 and 6 deletions and inversions, respectively) and chromosome 26 has the lowest number of duplications (7). By contrast, BTA5, BTA18 and BTA3 showed the highest number of deletions, duplications and inversions, respectively (S2 Table).

**Fig 3:**
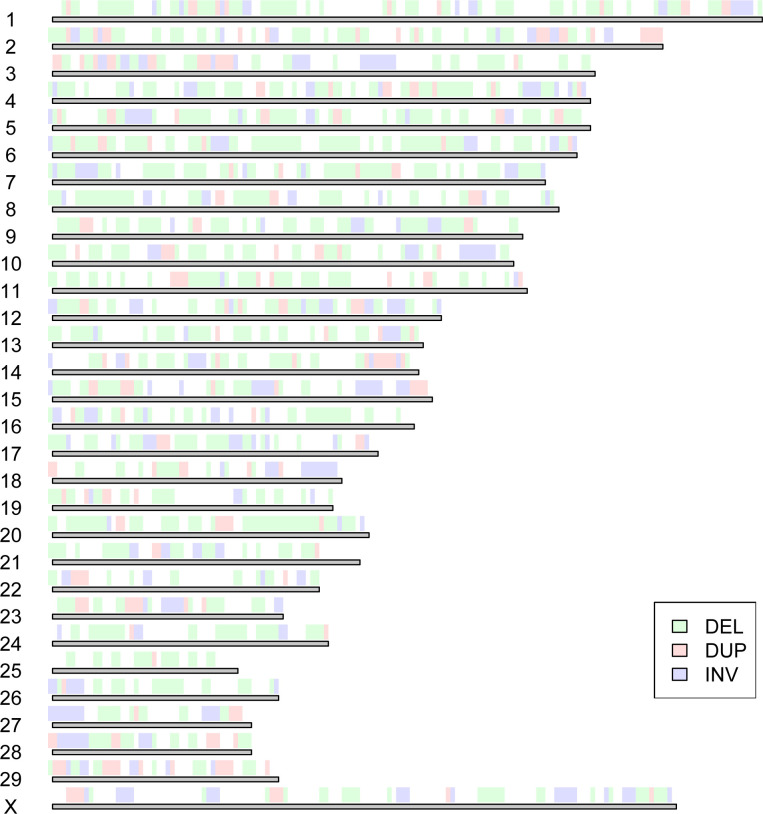
Chromosomal distribution of nonredundant SV regions with MAF > 0.25 and size > 1 Kb.

Overall, common and highly frequent SV (MAF > 0.25) detected in the present study covered a total length of ~ 446 Mb which corresponds to almost 17% of the ARS-UCD1.2 assembly. Analysis of the distribution of SV regions across chromosomes showed substantial variation depending among others on SV type (Fig 3). Chromosome-wide SV coverage along each chromosome varies from 6.36% on chromosome 25 to 39.4% on chromosome 28 (S1 Fig) and S3 Table) and was not correlated to the number of SV regions (S3 Table). BTX is the most densely covered with SV in terms of Megabases (32 Mb) with inversions representing 70% of its whole SV length (S3 Table). Similar finding was reported by [44]. They explained this by difficulties when mapping SV on the X chromosome especially in males where effective coverage is halved. Another possible explanation for the observed higher number of SV observed on the X chromosome in the 23 Creole cattle bulls is likely related to the high male specific contribution of the X-chromosome to individual global recombination rate, previously reported in several cattle breeds [45]. It is therefore reasonable to assume that chromosomes with higher recombination rates are more likely to show more SVs since these are generated by different recombination mechanisms.

We identified 6065, 464 and 343 new deletion, duplication and inversion regions, respectively that have not been previously reported in the DGVa database. Together, the three types SV defined 6638 nonredundant regions that have not been previously reported in DGVa (S4 Table). Focusing on SV > 1 Kb, 1967, 238 and 221 new deletion, duplication and inversion regions, were detected respectively (S2 Fig). Among these, three deletions, located on chromosomes 12, 16 and 26 and three duplications, located on chromosomes 7, 28 and X, are highly frequent in the GUA sample (S5 Table).

### Annotation of SV

**Fig 4:**
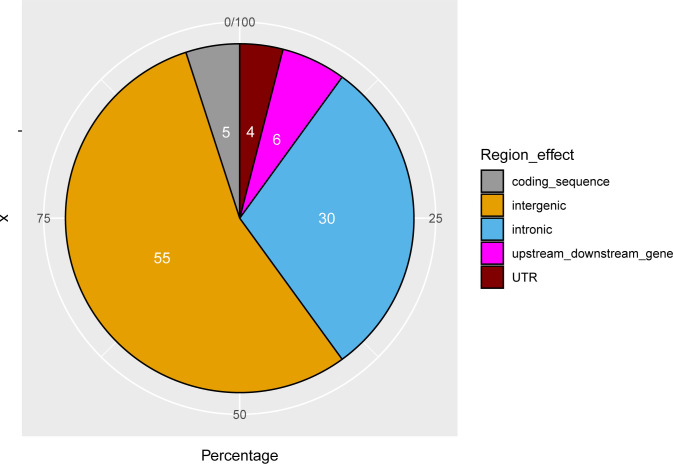
Variant effect predictor output from the genomic regions defined by nonredundant SV with size > 1 Kb.

Structural variants that occur in genes can alter gene expression either by changing gene dosage or interrupting coding sequences, or disturbing long-range gene regulation which could broadly influence phenotypes. To better predict the downstream effect of the detected SV on protein function, we used the Ensembl VEP tool [46]. To get potential important insights into population-level effect of SV on genes with adaptive functions in cattle breeds raised under tropical environment, we merged the common and highly frequent SV into a set of nonredundant 9734 SV regions among which 55% were intergenic (Fig 4). SV regions occurring in genic regions overlapped with 7793 genes and 12,922 transcripts. According to VEP, almost all of the detected SV were assigned to the “modifier” impact category which is not surprising since most of these SV were located within introns (Fig 4). Only three deletions, located on BTA8 (at positions: 9,754,093–9,839,766), BTA10 (at position:101,090,067–101,094,332 bp) and BTA15 (at position: 45,917,286–45,918,286 bp), were classified as having a high (disruptive) impact on the protein function. Three genes are affected by these SV: *HMBOX1*, *FOXN3* and a novel cattle gene: *ENSBTAG00000027525*. The first two genes are transcription factors. *HMBOX1* is a transcriptional repressor that negatively regulates IFN-γ in natural killer cells [47]. While IFN-γ production is momentarily abrogated following intense exercise which provides a window for invasion of pathogens [48], the deletion in *HMBOX1* might be regarded as a compensation mechanism that substitutes the downregulation of *IFN-γ* during prolonged exhausting exercise. Creole cattle from Guadeloupe are mainly used for a draught of sugar cane carts or ploughing [49] which would weaken their immune system for large parts of the day. *HMBOX1* might have also a protective role against splenomegaly and anaemia, which are prominent features of trypanosomiasis in cattle [50]. Unlike *HMBOX1*, the adaptive interpretation of *FOXN3* deletion (observed in nearly 83% of our GUA sample), is less obvious because this gene is involved in a variety of physiological processes ranging from cell proliferation, apoptosis and pathogenesis in human cancer [51] to craniofacial development and fasting blood glucose and glucagon, in other non-human species [52–54]. In Angus cattle, it has been associated with chest width and skeletal development [55]. Further investigations of the functional effects of partial deletions *FOXN3* on adaptive features of cattle are needed.

We also checked if there is any effect of SV frequency on their functional consequences by comparing common and highly frequent SV on one hand and low frequency SV on the other hand (these are defined as SV detected in less than four individuals). We found that SV of the second category tend to be in coding sequences more often than the first category (738 Vs 487, χ-squared = 51.712, p-value = 6.425 x 10^−13^).

We then generated a gene list including 579 genes that overlap with the genomic regions specific to GUA population (those that were not reported in DGVa) and encompassing common and highly frequent deletions and duplications. These genes were annotated according to DAVID Bioinformatics resources (https://david.ncifcrf.gov/). We found significant enrichment of the functional category ‘UP_SEQ_FEATURE: DOMAIN:C2’ (Benjamini-corrected P-value = 0.034, n = 13). Calcium transport was the most enriched biological process (BP) term (Benjamini-corrected P-value = 0.0011, n = 10) (S6 Table). C2 domains are widespread and conserved motifs that often serve as Ca2+-binding modules. Single and multiple copies of C2 domains have been identified in a growing number of eukaryotic signalling proteins that interact with cellular membranes and mediate a broad array of critical intracellular processes, including membrane trafficking, the generation of lipid-second messengers, activation of GTPases, and the control of protein phosphorylation [56]. In total, thirteen C2 domain genes located on 11 different chromosomes were impacted by SV. Among these, eight genes: *CPNE4*, *PKN2*, *PIK3C2G*, *UNC13C*, *DYSF*, *PLCB1*, *SYT9*, *SMURF2* are affected by frequent deletions (affecting more than 65% of our GUA sample). In ruminants, several of these genes have previously been associated with growth traits, such as *CPNE4 or DYSF* [57,58] or with fat metabolism, such as *PLCB1* [59]. Interestingly, the differentiation of some of these genes between taurine and indicine cattle have also been discussed (*CPNE4*, *DYSF*, *PIK3C2G*) [57,58,60]. Three of these genes (*DYSF*, *PLCB1*, *SMURF2*) are also known to regulate many aspects of the immune system response to pathogens. Similar to *HMBOX1*, *SMURF2* and *DYSF* negatively regulate some aspects of immune response. *SMURF2* gene is an essential negative regulator of TGF-β signalling and plays a role in the vascular inflammatory response in the presence of hypoxia in endothelial cells [61] while *DYSF* negatively regulates phagocytosis (GO:0050765). Finally, *PKN2* appears essential for embryogenesis in mouse [62], and its loss causes severe cardiovascular and morphogenetic abnormalities. More research would be necessary to identify the impact of these deletions in cattle, in particular in GUA breeds.

We took a closer look at the three deletions and the three duplications that were not previously reported in the DGVa database and which are highly frequent in our GUA sample (S5 Table). These six CNV regions encompass 135 genes, most of them (67 genes) are located on chromosome X. We have focused primarily on genes for which there is evidence for local adaptation in GUA cattle. In this regard, we find that duplications on BTA28 encompass two genes, *ras homolog family member U* (*RHOU*) and *actin alpha 1*, *skeletal muscle (ACTA1)* that play a key role in muscle development and contraction [63–65]. *RHOU* also regulates cell-adhesion molecules during cardiac morphogenesis [66]. Creole cattle from Guadeloupe have often been used as a major labour force in sugarcane fields and are known for their draft endurance. Duplications in these genes might be a hint that allows physiological adaptation of GUA population to such strenuous activities. In humans, it has been shown that SV occurring in genes expressed in muscle or heart contribute to the variation of endurance capacity [67]. CNV observed in *RHOU* were also reported as involved in mammary development in Dairy Gir cattle [68]. Deletions on BTA26, overlaps with *INPP5A*, that have been associated with body temperature regulation in Nellore cattle, through the changes in the nervous system and regulation of inflammatory processes [69].

In other respects, we observed that almost all duplicated genes on BTA7 included olfactory receptors which are well known for their extremely frequent gene duplications and losses in vertebrates [70]. Population specific SV affecting olfactory receptor genes were also reported in cattle [71]. Likewise, we find that GUA-specific structural variants were also found to overlap with genes influencing milk as well as meat traits in cattle. For instance, duplications on BTA28 overlap with 15 genes, some of which (*RAB4A*, *CCSAP*, *ENSBTAG00000048654*, *or URB2*) have previously been associated with fat or protein metabolism involved in beef or milk production traits [72,73]. The same finding holds for *GPC6*, *TGDS*, *GPR180* and *SOX21* genes which overlap with deletions on BTA12 [72,74].

The 135 genes were analysed for GO term enrichment. Gene Ontology (GO) analysis showed that tRNA threonylcarbamoyladenosine metabolic process is the most enriched biological process (BP) term (GO:0070525, n = 4, Benjamini-corrected p-value = 7.03× 10^−4^). tRNAs are central players in translation, functioning as adapter molecules between the informational level of nucleic acids and the functional level of proteins. Modifications on tRNA structure modulate rigidity and flexibility of the transcripts and confer thermal adaptation in thermophilic as well as psychrophilic bacteria [75]. Although tRNA modifications play multi-faceted roles in several cellular processes, they remain largely unexplored in mammals. In humans, analysis of tRNA indicates that many tRNA modifications are incomplete under physiological conditions and that variation in the levels of tRNA modification should enable cellular adaptation to environmental changes [76].

### Overlap between structural variants and previously identified signatures of selection in the GUA genome

We investigated the potential overlap between the highly frequent structural variants, identified in the present study and six genomic regions that have been recently shown to be under selection in the GUA population [25]. These six regions are located on BTA2 (at position: 120–120.5 Mb), BTA4 (at position: 113–113.5 Mb), BTA5 (at position: 47–47.5 Mb), BTA6 (at position: 69–69.5 Mb), BTA12 (at position: 29–30 Mb) and BTA13 (at position: 63.5–64 Mb). Within each region, we identified one or two candidate genes, based on their involvement in adaptive traits: *EIF4E2* (CR on BTA2), *GIMAP* genes (CR on BTA4), (*GRIP1* and *HELB* on BTA5*)*, *LNX1* and *OCIAD1* (CR on BTA6), *RXFP2* (CR on BTA12) and *ASIP* (CR on BTA13).

Overall, the six candidate regions included 17 structural variants ranging between 50 bp and 253.7 Kb in length (14 deletions and 3 inversions). *GIMAP* genes (*GIMAP4 –GIMAP7*) are located within an inversion of 253 Kb which overlapped with a previously reported CNV (nsv616158, [43]) in cattle (S7 Table). They are related to the primary immunodeficiency pathway and were also shown to play a major role in feed utilization and the metabolism of lipids, sugars, and proteins in Jersey cattle, and present a signature of selection in Asturiana de los Valles Spanish breed [77,78]. Among the SV specific to GUA individuals, we identified two highly frequent deletions of 50 bp and 1056 bp in length affecting the intronic regions of *HELB* and *GRIP1*, respectively. *HELB* is involved in the response to DNA damage including exposure to ultra-violet light and specific mutations carried out by indicine cattle and admixed populations have already been described. It appears to be associated with reproductive traits and yearling weight in tropical cattle, and could contribute to the adaptation of tropical cattle to their harsh environment [79]. Likewise, we found that a 5.5 Kb-length deletion (at position:120,098,203–120,103,733 bp) on chromosome 2 that affects a coding sequence of *ALPI* gene and have a high predicted functional impact on the protein (S7 Table). This gene is involved in diverse biological processes, including modulating host-bacterial interactions [80], mucosal defence and maintaining gut homeostasis [81]. Functional studies would need to be conducted to investigate whether the identified deletion impacts the adaptive immune system of GUA cattle which in turn shapes the diversity and the balance of gut microbiota required for immune homeostasis.

On chromosome 6, a deletion affecting 18 out of 23 GUA individuals is present in *LNX1* gene, a major regulator of the presynaptic glycine transporter *GlyT2* [82]. The 334-bp deletion in our sample overlaps an intronic region and have a “modifier” impact, which supposes that it has a mild effect on phenotypes in GUA cattle. Importantly, mutations in the exon 4 of GlyT2 were shown to cause recessive congenital muscular dystonia type 2 (CMD2) in Belgian Blue cattle [83]. An association between *LNX1* and birth weight has been reported in the Colombian creole breed Blanco Orejinegro [84]. In humans, mutations in GlyT2 gene are responsible hyperekplexia, a condition in which affected individuals have increased muscle tone (hypertonia) and an exaggerated startle reaction to unexpected stimuli (tactile or acoustic) [85]. The impact of this deletion in GUA cattle would be interesting to evaluate, whether it affect embryo development, muscle tone or behaviour.

The closest SV to *RXFP2* and *ASIP* (candidate genes on chromosomes 12 and 13, respectively) are two deletions located 11.5 and 14.5 Kb upstream *RXFP2* and *ASI*P, respectively. Importantly, we found another deletion on chromosome 13 held by ~ 50% of GUA individuals, affecting *EIF2S2* gene which is differentially expressed in the skin epidermis of Humans, thus playing potential role in pigmentation phenotypes [86]. Some other genes affected by SV in GUA are also associated with coat colour determinism in cattle such as *DCT*, *OCAID1 (*close to *KIT* gene) and *ASIP* [87–89] which may explain coat color variation observed in this breed.

## Conclusions

Characterizing the genomic patterns of structural variants in local livestock breeds could aid in identifying phenotypically relevant loci involved in environmental adaptation. This study presents the first whole genome sequencing-based description of structural variants within the Creole cattle from Guadeloupe. We show that SV are a major source of the genome diversity of GUA population. We found evidence that an SV with a high impact consequence is associated with the interaction between gut microbiota and host immune system in this breed and that several novel and previously identified SV may play a role in several GUA-specific adaptive traits such as immune response to pathogens, thermotolerance and physical endurance. Our study motivate further research to investigate the functional effects of the identified structural variants on adaptive and production traits in tropical cattle breeds, in particular the physiological impact of variants inherited from zebu.

## Supporting information

S1 Fig Percentage coverage of chromosomal length by structural variants. (EPS)

S2 Fig Circular map of the new SV regions identified in the GUA genome. (EPS)

S1 Table Summary of Creole cattle sequencing data. (DOC)

S2 Table Chromosomal distribution of common and highly frequent SV having a size > 1 Kb in the 23 GUA samples. (DOC)

S3 Table Chromosomal distribution and chromosome coverage of nonredundant SV regions. (DOC)

S4 Table Newly identified Svs (in comparison to those reported in the DGVa database). (XLSX)

S5 Table Chromosomal coordinates and gene content of the six newly identified SV (3 deletions and 3 duplications) showing high frequency in the GUA sample. (DOC)

S6 Table Functional annotation clustering results for candidate genes overlapping common and highly frequent new deletions and duplications (not previously reported in DGVa) and having a size > 1 Kb. Significantly enriched functional term clusters (Benjamin-corrected p-value < 0.05) are in bold.
(XLS)

S7 Table Overlap between the common and highly frequent structural variants (size >1 Kb) and the six candidate regions putatively under selection identified in GUA (Ben-Jemaa, personal communication). SV in bold are those that have not been previously reported in the DGVa database. The two last columns indicate the variant molecular consequences and its severity as predicted by the Ensembl Variant Effect Predictor (VEP).
(DOC)
