# Supplementary material for: Uncovering structural variants in Creole cattle from Guadeloupe and their impact on environmental adaptation through whole genome sequencing
Source: PLoS One. 2024 Aug 26;19(8):e0309411. doi: 10.1371/journal.pone.0309411 (PMC11346954; doi:10.1371/journal.pone.0309411)
Supplement: S1 Table — (DOC) [file pone.0309411.s003.doc]

S1 Table. Summary of Creole cattle sequencing data.

| Sample_ID | Reads | Mapped reads | Mapping rate | Mapping depth | Sequencer |
| --- | --- | --- | --- | --- | --- |
| COLA21710 | 321671142 | 308319167 | 95.85 | 16.08 | HiSeq3000 |
| COLA21705 | 185963860 | 178672611 | 96.08 | 9.3 | HiSeq3000 |
| 60021 | 190211772 | 181782828 | 95.57 | 10.34 | HiSeq3000 |
| 676643 | 272926004 | 262009771 | 96.00 | 13.65 | HiSeq3000 |
| 60024 | 419131126 | 401127757 | 95.70 | 20.96 | HiSeq3000 |
| COL035227 | 365286556 | 348179349 | 95.32 | 18.26 | HiSeq3000 |
| COL035226 | 213783080 | 204942141 | 95.86 | 10.69 | HiSeq3000 |
| COLA20307 | 294066572 | 280454863 | 95.37 | 14.7 | HiSeq3000 |
| COL035264 | 403254024 | 385860397 | 95.69 | 20.16 | HiSeq3000 |
| COL035265 | 407586686 | 391938496 | 96.16 | 20.38 | HiSeq3000 |
| COL035266 | 268908996 | 257354278 | 95.7 | 13.45 | HiSeq3000 |
| COL035363 | 343876754 | 328435914 | 95.51 | 17.19 | HiSeq3000 |
| COL352442 | 355475416 | 339372280 | 95.47 | 17.77 | HiSeq3000 |
| COLA20312 | 334272410 | 319796461 | 95.67 | 16.71 | HiSeq3000 |
| COL035228 | 368905906 | 353329064 | 95.78 | 18.45 | HiSeq3000 |
| COLA03320 | 227384038 | 219455771 | 96.51 | 11.37 | HiSeq3000 |
| COL035362 | 475309462 | 451229948 | 94.93 | 23.77 | HiSeq3000 |
| COL352443 | 320407738 | 306296236 | 95.60 | 16.02 | HiSeq3000 |
| COL352440 | 366763352 | 349201156 | 95.21 | 18.34 | HiSeq3000 |
| COLA20310 | 317963678 | 303253799 | 95.37 | 15.9 | HiSeq3000 |
| COLA20309 | 372901582 | 358180879 | 96.05 | 18.65 | HiSeq3000 |
| COLA20311 | 276537630 | 265481631 | 96.00 | 13.83 | HiSeq3000 |
| COL352441 | 413867220 | 396133238 | 95.72 | 20.69 | HiSeq3000 |
