# Supplementary material for: Uncovering structural variants in Creole cattle from Guadeloupe and their impact on environmental adaptation through whole genome sequencing
Source: PLoS One. 2024 Aug 26;19(8):e0309411. doi: 10.1371/journal.pone.0309411 (PMC11346954; doi:10.1371/journal.pone.0309411)
Supplement: S2 Table — (DOC) [file pone.0309411.s004.doc]

S2 Table. Chromosomal distribution of common and highly frequent SV having a size > 1 Kb in the 23 GUA samples.

| CHR | Deletions | Duplications | Inversions |
| --- | --- | --- | --- |
| 1 | 252 | 47 | 37 |
| 2 | 235 | 52 | 40 |
| 3 | 213 | 73 | 78 |
| 4 | 277 | 102 | 51 |
| 5 | 319 | 139 | 52 |
| 6 | 216 | 27 | 36 |
| 7 | 231 | 56 | 54 |
| 8 | 178 | 40 | 45 |
| 9 | 172 | 22 | 33 |
| 10 | 191 | 67 | 29 |
| 11 | 151 | 29 | 15 |
| 12 | 178 | 46 | 39 |
| 13 | 141 | 29 | 32 |
| 14 | 133 | 34 | 23 |
| 15 | 229 | 90 | 61 |
| 16 | 134 | 42 | 23 |
| 17 | 107 | 14 | 28 |
| 18 | 259 | 174 | 64 |
| 19 | 112 | 51 | 24 |
| 20 | 122 | 12 | 14 |
| 21 | 144 | 52 | 33 |
| 22 | 65 | 12 | 10 |
| 23 | 207 | 110 | 34 |
| 24 | 80 | 14 | 11 |
| 25 | 47 | 13 | 6 |
| 26 | 92 | 7 | 23 |
| 27 | 95 | 48 | 22 |
| 28 | 85 | 26 | 21 |
| 29 | 124 | 53 | 24 |
| X | 140 | 62 | 45 |
