# Supplementary material for: Uncovering structural variants in Creole cattle from Guadeloupe and their impact on environmental adaptation through whole genome sequencing
Source: PLoS One. 2024 Aug 26;19(8):e0309411. doi: 10.1371/journal.pone.0309411 (PMC11346954; doi:10.1371/journal.pone.0309411)
Supplement: S3 Table — (DOC) [file pone.0309411.s005.doc]

S3 Table. Chromosomal distribution and chromosome coverage of nonredundant SV regions obtained after merging overlapping common and highly frequent SV having a size > 1 Kb in the 23 GUA samples. The last row shows the Pearson correlation coefficient between the number of each type of SV region and SV coverage.

| CHR | N SV regions | SV length  (Mb) | SV coverage (%) | N DEL regions | DEL length | DEL_Coverage (%) | N DUP regions | DUP length (Mb) | DUP Coverage  (%) | N INV regions | INV_Coverage  (%) | INV length (Mb) |
| --- | --- | --- | --- | --- | --- | --- | --- | --- | --- | --- | --- | --- |
| 1 | 223 | 23.94 | 15.10 | 215 | 11.77 | 7.43 | 31 | 10.51 | 6.63 | 31 | 7.10 | 11.26 |
| 2 | 213 | 23.05 | 16.92 | 200 | 6.45 | 4.73 | 34 | 16.07 | 11.80 | 35 | 4.50 | 6.13 |
| 3 | 170 | 19.99 | 16.52 | 153 | 8.98 | 7.42 | 40 | 8.36 | 6.91 | 35 | 10.24 | 12.39 |
| 4 | 156 | 22.90 | 19.08 | 163 | 11.01 | 9.18 | 33 | 11.25 | 9.38 | 24 | 7.91 | 9.49 |
| 5 | 199 | 20.09 | 16.73 | 195 | 11.99 | 9.98 | 37 | 9.36 | 7.79 | 24 | 8.34 | 10.01 |
| 6 | 228 | 11.61 | 9.85 | 199 | 7.99 | 6.78 | 25 | 3.48 | 2.96 | 29 | 3.27 | 3.85 |
| 7 | 202 | 17.94 | 16.21 | 192 | 8.15 | 7.37 | 37 | 6.12 | 5.53 | 26 | 10.56 | 11.69 |
| 8 | 167 | 11.70 | 10.33 | 149 | 6.65 | 5.87 | 21 | 6.06 | 5.35 | 29 | 2.25 | 2.55 |
| 9 | 166 | 13.06 | 12.38 | 151 | 8.91 | 8.45 | 16 | 7.08 | 6.71 | 16 | 5.28 | 5.57 |
| 10 | 139 | 17.59 | 17.03 | 134 | 4.88 | 4.73 | 31 | 6.39 | 6.18 | 14 | 12.41 | 12.82 |
| 11 | 134 | 14.35 | 13.42 | 123 | 7.64 | 7.14 | 20 | 10.66 | 9.96 | 13 | 1.57 | 1.67 |
| 12 | 134 | 20.55 | 23.56 | 123 | 10.62 | 12.17 | 22 | 9.63 | 11.04 | 16 | 14.30 | 12.47 |
| 13 | 141 | 7.35 | 8.81 | 121 | 3.09 | 3.70 | 21 | 2.24 | 2.69 | 17 | 5.37 | 4.48 |
| 14 | 109 | 16.63 | 20.18 | 106 | 7.00 | 8.50 | 17 | 12.05 | 14.63 | 18 | 6.45 | 5.31 |
| 15 | 170 | 15.14 | 17.81 | 157 | 7.23 | 8.51 | 37 | 6.96 | 8.19 | 27 | 10.13 | 8.61 |
| 16 | 118 | 9.97 | 12.30 | 101 | 3.16 | 3.90 | 16 | 1.78 | 2.20 | 19 | 8.02 | 6.50 |
| 17 | 87 | 16.07 | 21.97 | 95 | 3.57 | 4.88 | 13 | 5.04 | 6.89 | 19 | 13.14 | 9.61 |
| 18 | 101 | 11.25 | 17.09 | 95 | 6.06 | 9.21 | 33 | 7.58 | 11.51 | 18 | 10.48 | 6.90 |
| 19 | 78 | 7.35 | 11.59 | 74 | 1.39 | 2.19 | 22 | 4.54 | 7.16 | 11 | 4.78 | 3.03 |
| 20 | 121 | 12.16 | 16.90 | 112 | 6.70 | 9.31 | 11 | 6.71 | 9.32 | 12 | 5.59 | 4.02 |
| 21 | 101 | 9.71 | 13.89 | 91 | 6.25 | 8.95 | 15 | 6.65 | 9.52 | 16 | 8.66 | 6.05 |
| 22 | 60 | 9.81 | 16.15 | 62 | 2.31 | 3.80 | 11 | 3.59 | 5.91 | 9 | 6.89 | 4.19 |
| 23 | 78 | 14.65 | 27.90 | 70 | 9.21 | 17.54 | 18 | 10.68 | 20.34 | 18 | 7.24 | 3.80 |
| 24 | 84 | 7.63 | 12.25 | 75 | 2.87 | 4.61 | 9 | 0.62 | 0.99 | 10 | 9.07 | 5.65 |
| 25 | 54 | 2.69 | 6.36 | 45 | 0.41 | 0.96 | 11 | 1.29 | 3.04 | 6 | 2.58 | 1.09 |
| 26 | 94 | 6.67 | 12.82 | 88 | 2.93 | 5.63 | 7 | 1.10 | 2.11 | 15 | 7.07 | 3.67 |
| 27 | 44 | 13.99 | 30.68 | 45 | 5.32 | 11.67 | 9 | 5.94 | 13.03 | 8 | 20.44 | 9.33 |
| 28 | 57 | 18.11 | 39.42 | 63 | 8.18 | 17.81 | 16 | 12.86 | 27.98 | 9 | 11.51 | 5.29 |
| 29 | 72 | 18.18 | 35.58 | 77 | 10.49 | 20.53 | 17 | 14.63 | 28.64 | 14 | 7.13 | 3.64 |
| X | 103 | 32.07 | 23.07 | 96 | 10.70 | 7.70 | 23 | 9.37 | 6.74 | 29 | 16.14 | 22.44 |
| Sum | 3803 |  |  | 3570 |  |  | 653 |  |  | 567 |  |  |
| r | -0.38 |  |  | -0.13 |  |  | -0.04 |  |  |  | -0.06 |  |
