# Supplementary material for: Uncovering structural variants in Creole cattle from Guadeloupe and their impact on environmental adaptation through whole genome sequencing
Source: PLoS One. 2024 Aug 26;19(8):e0309411. doi: 10.1371/journal.pone.0309411 (PMC11346954; doi:10.1371/journal.pone.0309411)
Supplement: S5 Table — (DOC) [file pone.0309411.s007.doc]

| S5 Table. Chromosomal coordinates and gene content of the six newly identified SV (3 deletions and 3 duplications) showing high frequency in the GUA sample. |
| --- |
| | Type | CHR | start | end | size(Kb) | protein coding genes | | --- | --- | --- | --- | --- | --- | | DEL | 12 | 67523104 | 69689400 | 2166.296 | *GPC6, ENSBTAG00000054671, ENSBTAG00000051263, DCT, ENSBTAG00000051519, TGDS, GPR180, SOX21, ENSBTAG00000032603* | | DEL | 16 | 47737652 | 48544435 | 806.783 | *-* | | DEL | 26 | 50595405 | 51154513 | 559.108 | *ENSBTAG00000049317, ADGRA1, ENSBTAG00000019919, ENSBTAG00000015651, NKX6-2, INPP5A, ENSBTAG00000054967* | | DUP | 7 | 9313855 | 10885278 | 1571.423 | *ENSBTAG00000052522, OR7A89, ENSBTAG00000055243, OR7A10, OR7A95, OR7A99, OR7A97, OR7A78, OR7A102, ENSBTAG00000047589, OR7A77, OR7A88, OR7A112, ENSBTAG00000053498, OR7A82, OR7A116, ENSBTAG00000052986, OR7A84, OR7A94, ENSBTAG00000052896, ENSBTAG00000055231, OR7A10B, OR7A91, OR7A96, ENSBTAG00000049961, ENSBTAG00000003528, ENSBTAG00000026148, ENSBTAG00000025908* | | DUP | 28 | 590528 | 3277126 | 2686.598 | *RHOU, RAB4A, CCSAP, ENSBTAG00000048654, ACTA1, NUP133, ABCB10, TAF5L, URB2, ENSBTAG00000053712, ENSBTAG00000011457, GALNT2, PGBD5, ENSBTAG00000048153, FMN2* | | DUP | X | 35267345 | 38216428 | 2949.083 | *VAMP7, SPRY3, TMLHE, CLIC2, ENSBTAG00000023978, RAB39B, ENSBTAG00000052586, VBP1, ENSBTAG00000011144, BRCC3, MTCP1, CMC4, FUNDC2, F8, ENSBTAG00000033167, MPP1, DKC1, GAB3, ENSBTAG00000023820, ENSBTAG00000020185, ENSBTAG00000047917, ENSBTAG00000050830, HAUS7, BGN, ATP2B3, CCNQ, DUSP9, PNCK, SLC6A8, BCAP31, ABCD1, PLXNB3, SRPK3, IDH3G, SSR4, PDZD4, ENSBTAG00000038379, L1CAM, AVPR2, ARHGAP4, NAA10, RENBP, HCFC1, TMEM187, IRAK1, MECP2, ENSBTAG00000052492, OPN1LW, TEX28, TKTL1, FLNA, EMD, RPL10, DNASE1L1, TAFAZZIN, ATP6AP1, GDI1, FAM50A, PLXNA3, LAGE3, UBL4A, SLC10A3, FAM3A, G6PD, ENSBTAG00000053534, IKBKG, ENSBTAG00000001900, ENSBTAG00000048914, ENSBTAG00000055292, ENSBTAG00000053848, ENSBTAG00000052652, ENSBTAG00000049584, ENSBTAG00000054052, ENSBTAG00000048281, ENSBTAG00000049678, ENSBTAG00000036343* | |
