# Supplementary material for: Uncovering structural variants in Creole cattle from Guadeloupe and their impact on environmental adaptation through whole genome sequencing
Source: PLoS One. 2024 Aug 26;19(8):e0309411. doi: 10.1371/journal.pone.0309411 (PMC11346954; doi:10.1371/journal.pone.0309411)
Supplement: S7 Table — SV in bold are those that have not been previously reported in the DGVa database. The two last columns indicate the variant molecular consequences and its severity as predicted by the Ensembl Variant Effect Predictor (VEP). (DOC) [file pone.0309411.s009.doc]

S7 Table. Overlap between the common and highly frequent structural variants (size >1 Kb) and the six candidate regions putatively under selection identified in GUA (Ben Jemaa et al., 2023). SV in bold are those that have not breen previously reported in the DGVa database. The two last columns indicate the variant molecular consequences and its severity as predicted by the Ensembl Variant Effect Predictor (VEP).

| Candidate region | SV start (bp) | SV end (bp) | SV type | SV length (bp) | Number of individuals | Candidate genes affected | Consequence | Impact |
| --- | --- | --- | --- | --- | --- | --- | --- | --- |
| 2 : 120,000,000 - 120,500,000 | 120098203 | 120103733 | DEL | 5531 | 20 | *ALPI* | stop_lost, coding sequence | high |
|  | **120272722** | **120274012** | **DEL** | **1291** | **23** |  |  |  |
| 4 : 113,000,000 - 113,500,000 | 113065181 | 113240690 | INV | 175510 | 21 | *GIMAP4 - GIMAP7* | intergenic | modifier |
|  | 113085262 | 113220575 | INV | 135314 | 20 |  |  |  |
|  | 113014377 | 113268123 | INV | 253747 | 18 | *GIMAP4 - GIMAP7* | intergenic | modifier |
| 5 : 47,000,000 - 47,500,000 | **47064355** | **47064438** | **DEL** | **82** | **22** |  |  |  |
|  | **47496192** | **47496243** | **DEL** | **50** | **19** | ***HELB*** | **intronic, truncation** | **modifier** |
|  | **47283473** | **47284528** | **DEL** | **1056** | **22** | ***GRIP1*** | **intronic, truncation** | **modifier** |
| 6 : 66,000,000 - 69,500,000 | **66918239** | **66920344** | **DEL** | **2106** | **18** |  |  |  |
|  | 68015547 | 68015637 | DEL | 89 | 23 |  |  |  |
|  | **68694901** | **68695146** | **DEL** | **244** | **23** |  |  |  |
|  | **68785370** | **68786236** | **DEL** | **867** | **19** |  |  |  |
|  | **69084617** | **69084950** | **DEL** | **334** | **18** | ***LNX1*** | **intronic, truncation** | **modifier** |
|  | 69402203 | 69404635 | DEL | 2433 | 19 |  |  |  |
| 12 : 29,000,000 - 30,000,000 | 29154772 | 29155546 | DEL | 775 | 20 |  |  |  |
|  | **29200689** | **29200878** | **DEL** | **190** | **20** |  |  |  |
| 13 : 63,500,000 - 64,000,000 | **63639817** | **63648209** | **DEL** | **8393** | **22** |  |  |  |
